# Supplementary figures and images for: The alteration of uterine microbiota participated in the activation of the decidual inflammatory response in early spontaneous abortion
Source: PLoS One. 2025 Feb 24;20(2):e0317595. doi: 10.1371/journal.pone.0317595 (PMC11849897; doi:10.1371/journal.pone.0317595)

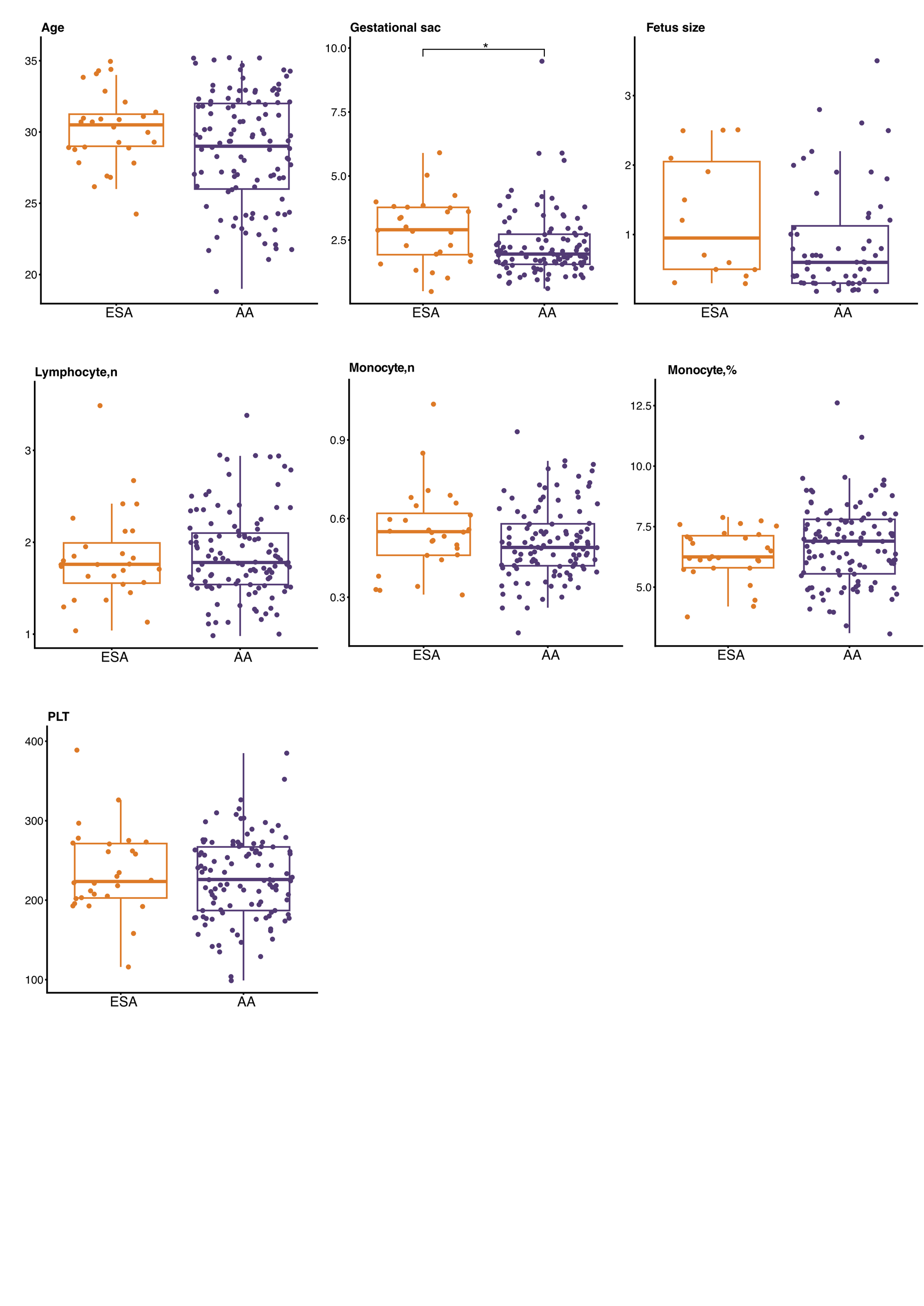

Supplement: S1 Fig — Comparison of the number of mean age, gestational sac, fetus size, number of lymphocytes, number of platelets, the number and percentage of monocyte between ESA and AA groups. Each dot represents an individual ESA (red), AA (blue). Significance determined by Student’s t test: * p < 0.05. ESA, early spontaneous abortion; AA, artificial abortion. (TIF) [file pone.0317595.s001.tif]

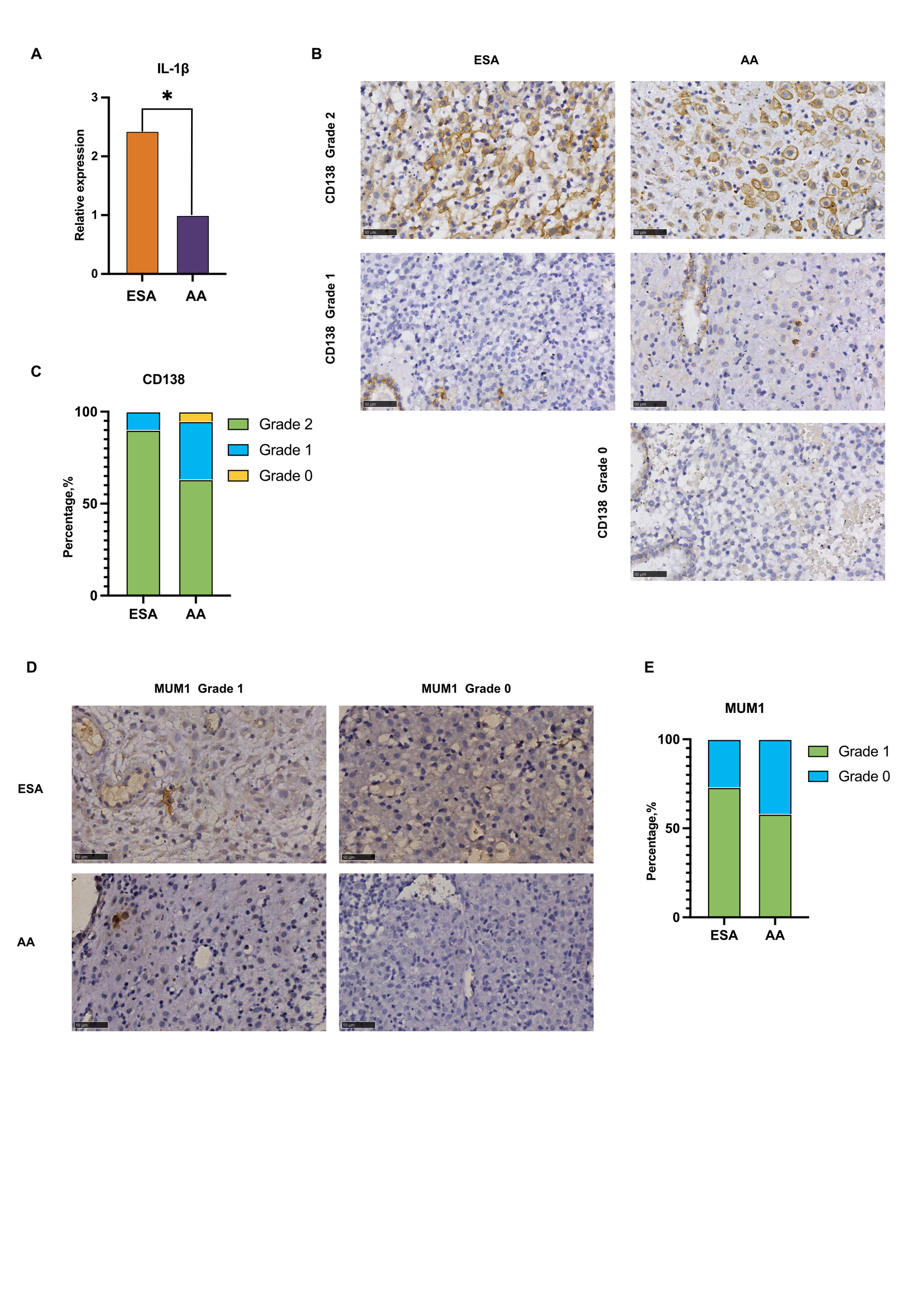

Supplement: S2 Fig — (A) Comparison of IL-1β RNA expression between ESA and AA by qPCR. (B) Immunohistochemical (IHC) staining and Grading of CD138 in decidua. (C) Distribution of different grade of CD138 staining between ESA and AA. (D) Distribution of different grade of MUM1 staining between ESA and AA. (E) Immunohistochemical (IHC) staining and Grading of MUM1 in decidua. Significance determined by chi2 test and Student’s t test: * p < 0.05. ESA, early spontaneous abortion; AA, artificial abortion. (TIF) [file pone.0317595.s002.tif]

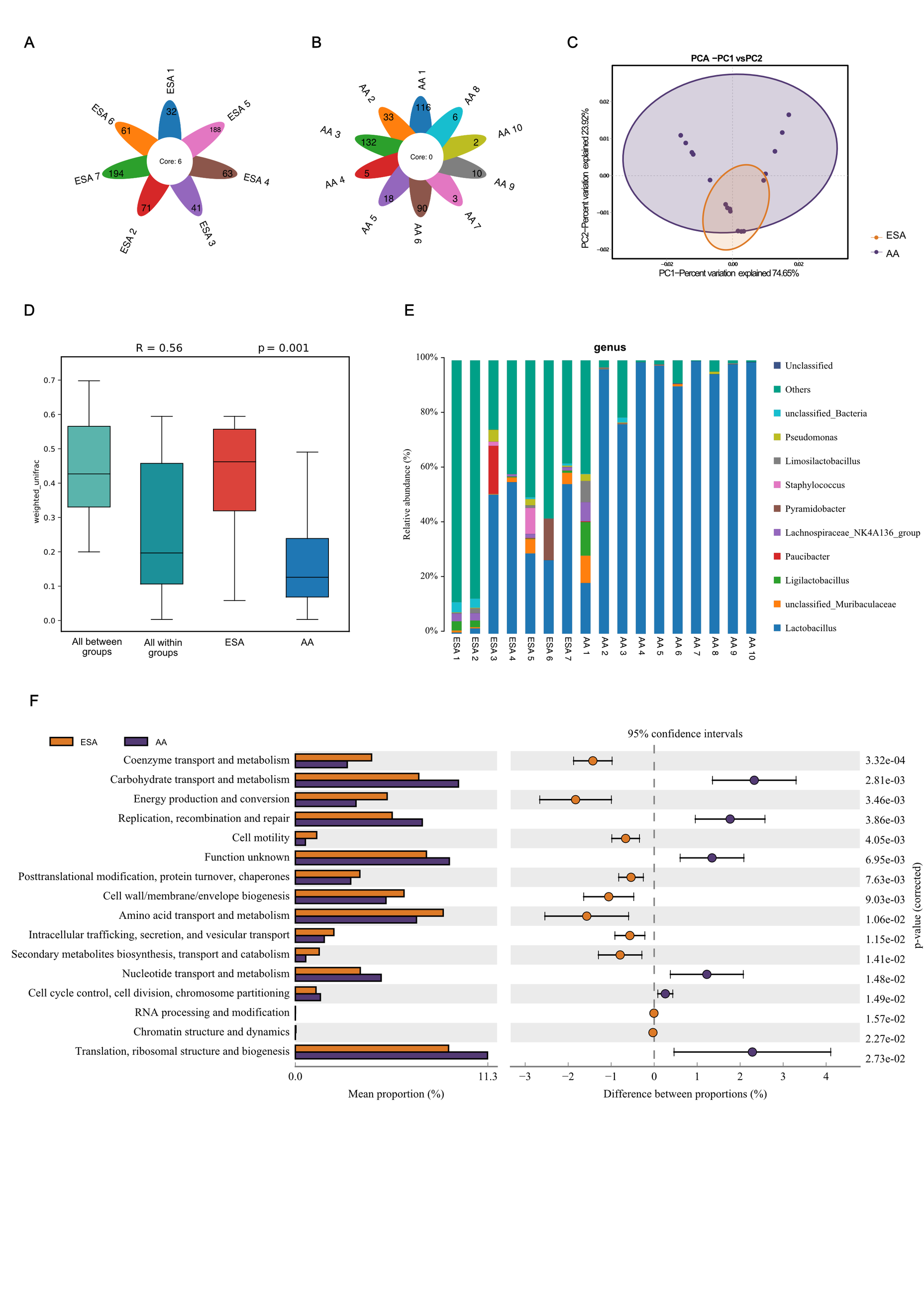

Supplement: S3 Fig — (A) and (B) OTUs analysis of each sample and group by petal diagram, the central circle represented the number of OTUs common in the same group, and the non-overlapping part was the number of OTUs unique to each sample. (C) PCoA plot of weighted-unifrac distance between ESA and AA groups. (D) Beta diversity by Anosim analysis of weighted-unifrac distance within and between ESA and AA groups. (E) Relative abundances of the uterine microbiota of each sample at the genus level. (F) Clusters of Orthologous Groups of proteins (COG) functional comparison of significantly differentially abundant bacterial in ESA and AA group. ESA, early spontaneous abortion; AA, artificial abortion. (TIF) [file pone.0317595.s003.tif]
