# Supplementary material for: The alteration of uterine microbiota participated in the activation of the decidual inflammatory response in early spontaneous abortion
Source: PLoS One. 2025 Feb 24;20(2):e0317595. doi: 10.1371/journal.pone.0317595 (PMC11849897; doi:10.1371/journal.pone.0317595)
Supplement: Attachment — Submitted filename: Figures.docx [file pone.0317595.s006.docx]

**Fig 1. The flowchart of patient cohorts in study**

*ESA, early spontaneous abortion; AA, artificial abortion*

**Fig 2. Clinical characteristics analysis of patient cohorts**

(A) Comparison of the number of leukocytes, percentage of lymphocytes, the number and percentage of neutrophils between ESA and AA groups. Each dot represents an individual ESA (red), AA (blue).

(B) Spearman correlation and hierarchical clustering of indicated features for ESA and AA groups.

Significance determined by spearman correlation test and student’s t test: *p < 0.05, **p < 0.01 and ***p < 0.001. *ESA, early spontaneous abortion; AA, artificial abortion*

**Fig 3. Analysis of inflammation cytokines expression of decidua between the ESA and AA groups.**

(A) Spearman correlation and hierarchical clustering of clinical features and 40 inflammation cytokine expression in decidua for ESA and AA groups.

(B) Heatmap of the representative cytokines expression in decidua between ESA and AA groups.

(C) Volcano plot showed the significantly upregulated or downregulated cytokines in decidua between ESA and AA groups.

(D) The immune-histochemistry staining of IL-1β in decidua of ESA and AA group.

(E) The comparison of IL-1β integrate optical intensity (IOD) between ESA and AA group.

(F) The double- immunofluorescence staining of IL-1β (green), prolactin (red) and DAPI (blue).

Significance determined by Spearman correlation test and Bayesian test with BH correction: *p < 0.05, **p < 0.01 and ***p < 0.001. *ESA, early spontaneous abortion; AA, artificial abortion*

**Fig 4. Analysis of the diversity and relative abundance of the uterine microbial composition in the ESA and AA groups**

(A) Venn diagram between ESA patient and AA groups at OTUs level.

(B) Hierarchical clustering heatmap of samples by weighted-unifrac algorithm.

(C) The comparison of alpha diversity between ESA patients and AA groups showed by Chao1 index.

(D) The comparison of alpha diversity between ESA patients and AA patients showed by Shannon index.

(E) The top10 relative abundance of different bacteria in the two groups at genus level. The middle is the difference in the abundance of bacterial groups within the 95% confidence interval; the right side is the P value, P<0.05 means the difference is statistically significant.

(F) The top5 linear discriminant analysis (LDA) scores obtained from the linear discriminant analysis effect size (LEfSe) analysis of the uterine microbiota in the two groups. Significance determined by Student t test or Wilcox rank sum test: *p < 0.05, **p < 0.01 and ***p < 0.001. *ESA, early spontaneous abortion; AA, artificial abortion*
