# Supplementary material for: The alteration of uterine microbiota participated in the activation of the decidual inflammatory response in early spontaneous abortion
Source: PLoS One. 2025 Feb 24;20(2):e0317595. doi: 10.1371/journal.pone.0317595 (PMC11849897; doi:10.1371/journal.pone.0317595)
Supplement: Attachment — Submitted filename: Response to Reviewers.docx [file pone.0317595.s007.docx]

Meng Cheng, Ph.D.

Department of Gynecology and Obstetrics

West China Second University Hospital of Sichuan University

1416, Section 1, Chenglong Avenue, Chengdu, 610066, China

E-mail: ttt-cheng@163.com

Tel: (86)-28-88570307

December 10^th^, 2024

Dear Editor and Reviewers,

Thank you all for your their positive and constructive feedback concerning our manuscript entitled “The alteration of uterine microbiota participated in the activation of the decidual inflammatory response in early spontaneous abortion” (PONE-D-24-35956). These comments are all valuable and very helpful for revising and improving our paper, as well as the important guiding significance to our research.

We have studied comments carefully and have made correction in the hope that they will meet with approval. Revised portions are highlighted in the paper. The corrections in the paper according to the responds to the requirements of the Journal and reviewer's comments are listed later in this letter.

Once again, we would like to acknowledge and thank the editors and reviewers for their valuable suggestions. We look forward to hearing from you regarding our submission. We would be glad to respond to any further questions and comments that you may have.

Kind regards and Merry Christmas.

Sincerely Yours,

Meng Cheng, Ph.D.

Department of Gynecology and Obstetrics

West China Second University Hospital of Sichuan University

**Journal Requirements:**

**1. Please ensure that your manuscript meets PLOS ONE's style requirements, including those for file naming.**

We checked our manuscript and confirmed its compliance with the PLOS ONE's style requirements.

**2. We note that the grant information you provided in the ‘****Funding Information’ and ‘Financial Disclosure’ sections do not match.**

We misunderstood ‘Financial Disclosure’ to corporate sponsorship. So, in the ‘Funding Information’ section we have filled in the official funding information. In the ‘Financial Disclosure’ section we put None. Following the reminder from the editor, the 'Funding Information' and 'Financial Disclosure' sections were revised and improved.

**3. Please state what role the funders took in the study.  Please include this amended Role of Funder statement in your cover letter; we will change the online submission form on your behalf.**

We revised the 'Funding Information' and 'Financial Disclosure' sections as following:

‘This research was funded by the National Natural Science Foundation of China (Website: <https://www.nsfc.gov.cn/>). MC received the grant (No.82101717). The funder had no role in study design, data collection and analysis, decision to publish, or preparation of the manuscript.’

In the revised cover letter, we also indicated the amended role of the Funder.

**4. Please review your reference list to ensure that it is complete and correct. If you have cited papers that have been retracted, please include the rationale for doing so in the manuscript text or remove these references and replace them with relevant current references. Any changes to the reference list should be mentioned in the rebuttal letter that accompanies your revised manuscript. If you need to cite a retracted article, indicate the article’s retracted status in the References list and include a citation and full reference for the retraction notice.**

We have verified that all the references are correct and have not been retracted. According to the reviewer's opinion, we read some recent relevant studies and add three references that we think very valuable. Below is the study we added:

1.Odendaal J, Black N, Bennett PR, Brosens J, Quenby S, MacIntyre DA. The endometrial microbiota and early pregnancy loss. Hum Reprod. 2024 Apr 3;39(4):638-646.

2.Edgar RC. Search and clustering orders of magnitude faster than BLAST. Bioinformatics (Oxford, England). 2010;26(19):2460-1.

3.Callahan BJ, McMurdie PJ, Rosen MJ, Han AW, Johnson AJ, Holmes SP. DADA2: High-resolution sample inference from Illumina amplicon data. Nature methods. 2016;13(7):581-3.

Responds to the reviewer's comments:

Reviewer #1:

**1. Figures and Tables: Figures are informative, but some (e.g., Figures 3 and 4) are dense and could benefit from being split into sub-panels for clarity. Explain the Bayesian test with BH correction in the figure legend.**

We have addressed your feedback by splitting the original Figure 3 into two separate figures (now Figures 3 and 4) for improved clarity. Additionally, the font size in the original Figure 4 (now Figure 5) has been adjusted to enhance readability.

Significance was assessed using Spearman’s correlation test and Bayesian analysis. For Bayesian tests, the Benjamini-Hochberg correction was applied to control the false discovery rate across multiple comparisons. Corrected results indicate the strength of evidence after adjusting for multiple hypotheses

**2 Methods:**

**2.1 Contamination Control: Provide more details on how contamination from the lower genital tract was controlled during 16S rRNA gene sequencing.**

We've added the following to the section of method.

The sample collection was conducted in a sterile operating room during the surgical termination of pregnancies. The patients were positioned in the lithotomy position. To prevent contamination from the vagina and cervix, the operator proceeded to sterilize the vagina and cervix, wipe away any cervical secretions, and dilate the cervical canal in accordance with the surgical procedure. Prior to the collection of the microbiota sample, the cervical canal was disinfected with iodophor and the residual iodophor was removed with a sterile dry cotton swab. Two new sterile dry cotton swabs were separately inserted into the uterine cavity to obtain the microbiota and then stored in a sterilized tube at -80°C prior to DNA extraction.

**2.2 Methods: Cytokine Assays: Clarify the use of negative controls in cytokine detection.**

The decidual endometrium represents the transformation of the endometrium that occurs following pregnancy because of the action of sexual hormones. In the gestational state, normal pregnancy and spontaneous abortion were used as controls for each other. Accordingly, the decidual endometrium of the AA group, which represents a normal pregnancy, and the ESA group were selected for study. In this instance, it was not feasible to implement a negative control.

**2.3 Methods: Patient Selection: Specify the randomization method used for selecting ESA and AA patients.**

This is single-center clinical observational study, not a randomized controlled study.

**3 Study Design:
3.1 Confounders: Clarify if lifestyle, diet, or other factors that affect microbiota were controlled.**

Thank you for your question. Since our study focuses on the endometrial microbiota, which is less influenced by daily diet and lifestyle compared to gastrointestinal microbiota, we did not specifically control for dietary and lifestyle factors. However, to ensure greater rigor in future studies, we plan to incorporate screening and control for these variables.

**3.2 Longitudinal Studies: Consider a longitudinal design to track microbiota and cytokine changes over time.**

We would like to express our gratitude to the reviewers for their valuable suggestions. In the future, we will give this matter further consideration.

**3.3 Mechanistic Studies: Future work could explore how microbial shifts drive inflammatory responses.**

We acknowledge and appreciate the valuable suggestions provided by the reviewers. The investigation of the mechanism including how microbial shifts drive inflammatory responses will be a principal focus of our future research.

**4 Statistics:
4.1 Multiple Comparisons: Clarify if corrections (e.g., Benjamini-Hochberg) were applied for multiple tests.**

Yes, the Benjamini-Hochberg correction was applied to control the false discovery rate across multiple comparisons

**4.2 Missing Data: Explain how missing data were handled, especially in microbial sequencing and cytokine profiles.**

This was a single-center clinical observational study, and we could not predict the type and number of patients who would attend the clinic during the observation time frame and whether they would be eligible for inclusion. In general, an equal number of AA and ESA groups should be included as far as possible. However, since the ESA group was sampled with no information on whether embryo chromosome abnormalities were present or not.

Therefore, when the embryonic chromosome results were available, some samples in the ESA group were excluded because of embryonic chromosome abnormalities. This resulted in the actual inclusion of 11 ESA and 19 AA patients (We already added this in the manuscript Line 277-280.). The samples from these patients were all used for inflammatory cytokine analysis, microbial sequencing and histological analysis. When 16S rRNA gene sequencing was performed, four uterine swab samples from the ESA group and nine uterine swab samples from the AA group were excluded because of DNA extraction failure (We have a description of it in the manuscript Line 307-308.). Additionally, we recognized the limitations of the data set in this section. In future studies of similar design, we will strive to reduce the proportion of missing data by increasing the sample size, extending the observation period and optimizing the sampling techniques employed.

**4.3 Confounding Variables: Describe whether multivariate analyses were performed to control for confounders.**

We thank you for your insightful suggestion. Multivariate analyses were performed using a logistic regression model, which inherently controlled for confounding variables by including some predictors (age, leucorrhea cleanliness, leucorrhea cell content, and leucorrhea lactobacillus content) in the same model. This approach allowed the estimation of the effect of each variable on the binary outcome while accounting for the influence of other included variables. The results, presented in **S1 Table**, demonstrated the adjusted associations, indicating that the observed effects were controlled for potential confounding among the predictors. (We already added this in the manuscript Line 251-259.)

**5 Results and data accessibility
5.1 Patient-Level Data: Include more granular patient-level data in supplementary materials to strengthen the correlation between microbiota and inflammatory responses.**

We acknowledged for your thoughtful critiques. Granular patient-level data were not included in the current analysis due to limitations in sample size for certain subgroups, which resulted in large statistical variances and potential bias in the tests. Stratified analyses revealed that some groups had insufficient data to produce robust and reliable results. To address this limitation, future research will aim to include a larger and more diverse sample size, enhancing the ability to detect meaningful correlations between microbiota and inflammatory responses. Expanding the dataset will improve statistical power and ensure more reliable subgroup analyses, strengthening the findings in subsequent studies.

**5.2 Outliers: Clarify how outliers in cytokine levels or microbiota data were handled.**

Thank you for raising this question. We reviewed the inclusion criteria and baseline characteristics of the outlier samples, confirming that they met the study standards. During the statistical analysis, normalization and standardization were applied to ensure the reliability of the data. Considering the sample size and the authenticity of the study conclusions, we chose to retain these samples in the analysis. We believe this approach provides a more comprehensive representation of the study population.

**6 References: Include recent studies on reproductive tract microbiota to provide context, and reference the bioinformatics tools (e.g., USEARCH, Silva database) more thoroughly.**

We thank for the careful review. We read some recent relevant studies and add three references that we think it very valuable. Below is the study we added:

1.Odendaal J, Black N, Bennett PR, Brosens J, Quenby S, MacIntyre DA. The endometrial microbiota and early pregnancy loss. Hum Reprod. 2024 Apr 3;39(4):638-646.

2.Edgar RC. Search and clustering orders of magnitude faster than BLAST. Bioinformatics (Oxford, England). 2010;26(19):2460-1.

3.Callahan BJ, McMurdie PJ, Rosen MJ, Han AW, Johnson AJ, Holmes SP. DADA2: High-resolution sample inference from Illumina amplicon data. Nature methods. 2016;13(7):581-3.

**7 Title: Consider simplifying the title.**

Thank you for your valuable suggestion. We tried to simplify our title to “The alteration of uterine microbiota participated in early spontaneous abortion”. However, the original title enables the reader to capture the main content of the text through the title. Thus, we kept our original title.

**8 Conclusions: Emphasize the need for future mechanistic studies to explain how microbiota shifts influence inflammation in ESA.**

We acknowledge and appreciate the valuable suggestions provided by the reviewers. The investigation of the mechanism including how microbial shifts drive inflammatory responses will be a principal focus of our future research. In future studies, we considered animal model studies, stratified patient analysis, control confounding factors and so on. Future work about mechanistic studies could explore how microbial shifts drive inflammatory responses.

**Reviewer #2:**

**1. Immunological interaction in the decidual environment is crucial in terms of pregnancy success. The concept has been clearly elucidated. One factor is the HLA-G expression in the trophoblastic cells and the detection of soluble isoforms can guide predicting pregnancy outcomes (pre-eclampsia is one of the causes of spontaneous abortion). Below is a study worth citing:
Bhattarai A, Shah S, Dahal K, Neupane R, Thapa S, Neupane N, Barboza JJ, Shrestha A, Sah R, Apostolopoulos V. Biomarker role of maternal soluble human leukocyte antigen G in pre-eclampsia: A meta-analysis. Immun Inflamm Dis. 2024 Apr;12(4):e1254. doi: 10.1002/iid3.1254. PMID: 38639563; PMCID: PMC11027746.**

We would like to express our gratitude to the reviewers for their valuable suggestions. We have read the literature carefully. The research content involved in this paper can be the focus of our future research. In the future, we will give this matter further consideration.

2 When interrogating in the introduction, I would like to request authors to mention that their objective was to answer those questions, rather than simply making interrogations.

We have raised two questions to clarify the purpose of our study and to engage the reader. Based on these two questions, we briefly describe the methodology and purpose of this study in the introduction. The answers and elaborations are presented in the discussion section.

Additionally, as the journal’s requirements, we uploaded all images to the Preflight Analysis and Conversion Engine (PACE) digital diagnostic tool (https://pacev2.apexcovantage.com/). The final images downloaded from the website were uploaded with the submission, along with the table of modifications generated by the tool.
